# Effects of atrial fibrillation on cerebral perfusion and cognitive function: A systematic review

**DOI:** 10.1016/j.ijcha.2026.101891

**Published:** 2026-02-20

**Authors:** Pernille Borch, Stine Aagaard-Nilsen, Mathis Korseberg Stokke, Erik Lyseggen

**Affiliations:** a Department of Cardiology, Oslo University Hospital Rikshospitalet, Oslo, Norway; b Institute for Experimental Medical Research, Oslo University Hospital and University of Oslo, Oslo, Norway

**Keywords:** Atrial fibrillation, Cerebrovascular circulation, Cognitive dysfunction, Heart failure

## Abstract

Atrial fibrillation (AF) has been associated with cognitive impairment. Impaired cerebral perfusion is a proposed mechanism, though evidence remains inconclusive, particularly in patients with concomitant heart failure (HF). We conducted a systematic review in accordance with PRISMA 2020 guidelines. PubMed and EMBASE were searched from database inception to May 9, 2025 for adult studies comparing cerebral perfusion in AF and sinus rhythm (SR). Risk of bias was assessed using ROBINS-E and ROBINS-I; certainty of evidence was graded using GRADE. Twenty-three studies were included (11 interventional, 1 observational, 11 cross-sectional). Measurement methods and effect sizes were heterogeneous. Most studies had serious/critical risk of bias, and certainty of evidence was low. Twenty-two studies concluded that AF may impair cerebral perfusion, while one found no such effect. Nine studies reported cognitive testing, and five demonstrated impaired performance in AF or improvement following SR restoration. In HF subgroup analyses (2 studies), perfusion was lower in HF patients with AF than those in SR. A third study found higher pulsatile index in HF patients with AF than those without HF, but no difference in peak, mean or diastolic cerebral blood flow velocities. Current clinical evidence suggests that AF may impair cerebral perfusion and contribute to cognitive decline. However, methodological limitations and heterogeneity limit the conclusions, and further longitudinal studies with standardized perfusion metrics are needed, including in concomitant HF where data are scarce.

## Highlights

• AF may impair cerebral perfusion and contribute to cognitive decline.
• Most studies found reduced brain perfusion in AF compared with sinus rhythm.
• AF was associated with impaired cognitive performance in multiple studies.
• Limited evidence suggests greater perfusion decline in AF with heart failure.

## Graphical abstract

Created in BioRender. BORCH, P. (2026) https://BioRender.com/yvbfpip. Abbreviations: AF, atrial fibrillation; HF, heart failure; SR, sinus rhythm; GRADE, Grades of Recommendation, Assessment, Development, and Evaluation.

**Figure:**
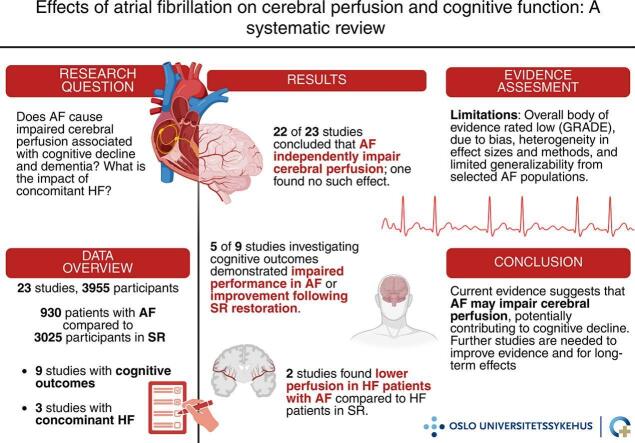

## 1 Introduction

Atrial fibrillation (AF) remains an important cause of morbidity and mortality worldwide, with a global prevalence of 59.7 million in 2019, which is expected to double in the coming decades [1]. While the elevated risks of stroke and heart failure (HF) in AF are well established, the rising prevalence, particularly among the older population, calls for increased attention also to lesser-known consequences [1]. Emerging evidence indicates that AF is independently associated with cognitive dysfunction and dementia, even after adjusting for stroke, comorbidities and anticoagulation therapy [2]. The mechanisms linking AF to cognitive decline remain unresolved but cerebral perfusion is often claimed to be a key contributor [3], [4]. One hypothesis states that the irregular heart rhythm in AF leads to variations in stroke volume and blood pressure that overwhelm the capacity of cerebral autoregulation, leading to repetitive hypo- and hyperperfusion events and a sustained state of impaired cerebral perfusion [2], [3]. Another hypothesis proposes that AF directly impairs cerebral autoregulation [5].

A recent systematic review of interventional studies concluded that cerebral perfusion improves following conversion from AF to sinus rhythm (SR) [4]. However, few studies are available, and a more comprehensive survey of available evidence was needed. Furthermore, it remains uncertain whether the findings assessed in the previous review extend to the broader AF population, particularly those with HF. This is an important point, as HF is present in approximately half of patients with AF [1]. HF might exacerbate the hemodynamic instability caused by AF, and thereby further impair cerebral perfusion [6], [7].

With this background, our objective was to assess total available evidence for whether AF, with or without concomitant HF, is associated with impaired cerebral perfusion, and that this contributes to cognitive dysfunction. For this, we performed a systematic review of interventional, observational and cross-sectional studies.

## 2 Methods

The study protocol adhered to the 2020 Preferred Reporting Items for Systematic Review and Meta-Analyses (PRISMA) guidelines [8], and was registered in the PROSPERO database (CRD42024501541). An explicit statement of the research question was made using the PICO framework:[9].

• **Problem:** Does AF cause impaired cerebral perfusion associated with cognitive decline and dementia?
• **Intervention:** Conversion from AF to SR.
• **Comparison:** (a) Within-patient comparison before and after successful conversion from AF to SR; (b) Between-patient comparison of successful vs unsuccessful conversion; and (c) Between-group comparison of patients with AF vs external control groups in SR (i.e. exposed vs unexposed).
• **Outcome:** The primary outcome was the impact of AF on cerebral perfusion metrics, assessed as either 1) “AF has an independent effect on cerebral perfusion”, or 2) “AF does not have an independent impact on cerebral perfusion”.

### 2.1 Search strategy

A systematic literature search was conducted in PubMed and EMBASE from inception to May 9, 2025.

#### 2.1.1 Search terms PubMed

(“Atrial Fibrillation”[MeSH] OR “atrial fibrillation”)

AND

(“Cerebrovascular Circulation”[MeSH] OR “Brain/blood supply”[Mesh] OR “Brain/metabolism”[Mesh] OR “cerebrovascular circulation” OR “cerebrovascular flow” OR “cerebral circulation” OR “cerebral perfusion” OR “brain perfusion” OR “cerebral blood flow” OR “brain blood flow” OR “cerebral microcirculation” OR “brain activity”)

#### 2.1.2 Search terms EMBASE

(“exp atrial fibrillation/” OR “atrial fibrillation.tw.”)

AND

(“exp cerebral blood flow/” OR “cerebral blood flow.tw.” OR “cerebrovascular flow.mp.” OR “exp brain circulation/” OR “brain circulation.tw.” OR “exp brain perfusion/” OR “brain perfusion.tw.” OR “exp brain microcirculation/” OR “brain microcirculation.tw.” OR “brain activity.mp.”)

#### 2.1.3 Filters Applied

“English language”: To enable first-hand evaluation of the original text by the authors.

“Human studies” (EMBASE): To avoid effects related to other species.

“Remove Medline records” (EMBASE): To minimize duplication of results with PubMed.

### 2.2 Eligibility criteria

Studies were eligible if they compared cerebral perfusion in AF and SR using any of the comparison approaches specified in the PICO framework (within-patient pre–post conversion, successful vs unsuccessful conversion, or AF vs SR control groups). Inclusion criteria comprised: 1) studies with data from patients > 18 years diagnosed with AF, 2) measurements of cerebral perfusion in SR and AF, 3) original research articles published in peer-reviewed journals, 4) published in English, 5) designed as a randomized controlled trial, observational study, case-control study or cross-sectional study.

Exclusion criteria were 1) studies without statistical or quantitative analyses directly comparing cerebral perfusion measurements in AF to those in SR, 2) publications reporting data derived from other publications, such as reviews or *meta*-analyses, and 3) reports, commentaries, letters, protocols, clinical guidelines and case reports.

In the context of cerebral hemodynamics, the terms “cerebral perfusion” and “cerebral blood flow” (CBF) are closely related but not identical. CBF typically refers to the measurable volume of blood delivered to a specific amount of brain tissue per unit time (commonly expressed as mL/100 g/min), whereas cerebral perfusion more broadly describes the adequacy of blood supply relative to metabolic demand. In this review, we used the term “cerebral perfusion” in a general sense, while acknowledging that many studies report direct measurements of CBF as a surrogate for perfusion. Across the included studies, cerebral hemodynamics were described using various terms including CBF, cerebral perfusion, and related concepts such as regional cerebral oxygenation. We accepted all studies measuring CBF or related metrics as indicators of cerebral perfusion, regardless of terminology. These variations were documented and considered in the data synthesis.

### 2.3 Study selection and data extraction

All papers from the PubMed and EMBASE search were transferred and stored in an EndNote library, where duplicates were removed. Two reviewers (P.B and E.L) independently screened titles and abstracts. Papers that both co-authors agreed met the inclusion criteria were selected for full-text review. In cases of discrepancy, the two reviewers discussed the studies to reach a consensus. If consensus could not be achieved, a third reviewer (M.K.S) was consulted.

Two reviewers (P.B and S.A.N) conducted a review of full-text articles, assessing them against the predefined inclusion and exclusion criteria. The following data from included studies were extracted using Covidence systematic review software [10]: Title, lead author contact details, journal, year of publication, aim of study, study type and characteristics (country, time and duration, funding sources, possible conflicts of interest), population description, definition of variables and primary and secondary outcomes.

The primary outcome was cerebral perfusion metrics, including but not limited to CBF velocity by transcranial doppler sonography, perfusion maps by magnetic resonance imaging or single-photon emission computed tomography, regional cerebral oxygen saturation by near-infrared spectroscopy and related indices. A p-value of < 0.05 was considered statistically significant.

### 2.4 Quality assessment

The included studies were evaluated by two reviewers (P.B and S.A.N) to assess the risk of bias using the ROBINS-E (Risk Of Bias In Non-randomized Studies of Exposures) tool for cross-sectional studies [11], and the ROBINS-I V2 (Risk Of Bias In Non-randomized Studies of Interventions Version 2) tool for intervention studies [12]. These tools were selected for their relevance to the study designs included in this review, enabling a comprehensive evaluation of potential biases including confounding, classification of interventions or measurement of exposure, patient selection, deviations from intended interventions or post-exposure interventions, missing data, outcome measurement and selective reporting of results. In accordance with ROBINS-I and ROBINS-E, the overall risk of bias was classified as 'critical' in instances where several domains were rated as having a 'serious' risk. As the tools do not define 'several' explicitly, we considered four or more domains with serious risk of bias as constituting a 'critical' overall rating. Discrepancies were resolved through discussion (P.B and S.A.N). If consensus was not reached, the two other reviewers (M.K.S and E.L) were consulted.

The overall strength of the body of evidence was eventually assessed based on the principles of the GRADE (Grades of Recommendation, Assessment, Development, and Evaluation) framework [13]. Quality of evidence was rated high, moderate, low, or very low, reflecting our certainty of the evidence.

### 2.5 Secondary outcomes and subgroup analysis

To explore the correlation between AF, cerebral perfusion and the development of cognitive decline or dementia, we collected data on cognitive testing from the studies that reported results from such tests.

To investigate individual and potential synergistic effects of AF and HF on cerebral perfusion, we specifically collected and assessed data from studies comparing cerebral perfusion in HF with and without AF, and AF with and without HF, including comparisons to healthy SR controls. Studies reporting subgroup analyses in patients with HF were included irrespective of left ventricular ejection fraction (LVEF). Accordingly, the term HF was defined to encompass all phenotypes, including reduced, mildly reduced, and preserved LVEF.

## 3 Results

### 3.1 Study selection

From the initial database search, 613 studies were identified. After removing 18 duplicates, 595 titles and abstracts were screened. Of these, 31 papers were eligible for full-text screening, while 564 studies were excluded due to irrelevant population, study design or publication type.

**Fig. 1:**
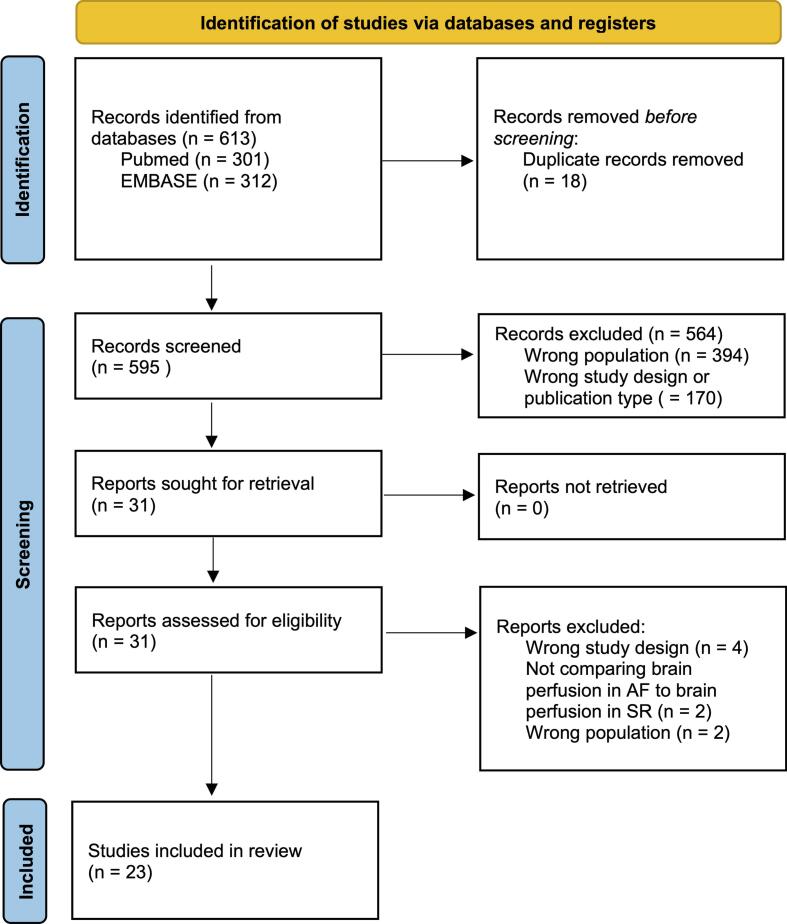
PRISMA flowchart showing the detailed steps of study selection [8].

After full-text screening, eight studies were excluded. Four did not measure cerebral perfusion, but focused on brain activity [14], [15], white matter changes and perivascular spaces on computer tomography [16], or volumes of hypoperfusion during stroke [17]. One study measured cerebral perfusion metrics in AF without comparisons to SR [18], one study reported perfusion metrics without statistical comparisons [19], and two studies focused on conditions other than AF [20], [21]. A total of 23 studies were included in the final analysis to evaluate the association between AF, cerebral perfusion, and related cognitive outcomes, as detailed in the following sections. The study selection process is shown as a PRISMA flowchart (Fig. 1).

### 3.2 Study characteristics

#### 3.2.1 Study designs

Of the 23 studies included, 11 were classified as pre-post interventional studies, one as an observational study without any intervention, and 11 as cross-sectional (Table 1). The pre-post studies measured cerebral perfusion before and after converting AF to SR. Interventions included electrocardioversion in five studies; catheter ablation in three; pharmacological conversion in two; and AV-node ablation followed by pacemaker implantation in one (Table 1). The last intervention, while not restoring SR, was included as it aims to achieve a normofrequent, regular ventricular rhythm despite continued AF, serving as a potential control for other AF-related effects. The observational study measured cerebral perfusion over a period during which patients spontaneously developed AF. Cross-sectional studies compared cerebral perfusion in AF patients versus controls in SR. All pre-post interventional studies included within-patient comparisons before and after conversion from AF to SR. Between-patient comparisons varied by study design. The heterogeneity of intervention methods in the pre-post studies allowed for multiple comparison groups (Table 1). In one study using catheter ablation, patients were in either AF or SR before the procedure and in either rhythm after the procedure, resulting in four distinct patient groups [22]. Four studies used patients with unsuccessful conversion as a control group for those with successful conversion [23], [24], [25], [26], one study used AF patients without any intervention [27], and four studies used non-AF participants in SR as external control groups [24], [25], [28], [29]. The observational study used within-patient comparisons before and after spontaneous occurrence of AF [30]. All cross-sectional studies used external control groups in SR, either healthy individuals or patients with other medical conditions such as hypertension or HF (see Section 4.2.2). Two cross-sectional studies included measurements during exercise [31], [32].

**Table 1:** **Characteristics of included studies**. Study groups are presented as rhythm status at baseline à follow-up cerebral perfusion measurements (pre-post studies), or rhythm status at time of assessment (cross-sectional studies). AF à SR indicates successful rhythm conversion and within-patient comparisons. AFà AF indicates unsuccessful conversion, (used as a control group when reported). In pre-post studies, SRà SR and SRà AF indicate patients with AF diagnosis undergoing intervention, who were in SR during baseline (e.g., paroxysmal AF), and remained in SR or were in AF at follow-up. AF controls are AF patients without rhythm intervention (used as a control group when reported). SR controls are external control participants in SR; when applicable, these are specified by comorbidity (e.g., HF SR, HT SR). **Abbreviations**: ABP, arterial blood pressure; ACE, angiotensin-converting enzyme; AD, Alzheimer’s disease; AF, atrial fibrillation; AFL, atrial flutter; AI, aortic insufficiency; aMCI, amnestic mild cognitive impairment; AMI, acute myocardial infarction; ARB, angiotensin receptor blocker; ASL, arterial spin labeling; AVLT, auditory verbal learning test; BMI, body mass index; BNP, brain natriuretic peptide; CA, catheter ablation; CABG, coronary artery bypass graft; CAD, coronary artery disease; CBF, cerebral blood flow; CCB, calcium-channel blocker; CCD, color-coded carotid duplex sonography; CHADS2, congestive heart failure, hypertension, age ≥75 years, diabetes mellitus, stroke or transient ischemic attack; CHA2DS2-VASc, congestive heart failure, hypertension, age ≥75 years, diabetes mellitus, stroke, vascular disease, age 65–74 years, and sex category; CHF, congestive heart failure; CI, cardiac index; CKD, chronic kidney disease; CRP, C-reactive protein; CTR, cardiothoracic ratio; CVR-CO2, Cerebrovascular CO2 reactivity; dBP, diastolic blood pressure; DFV, diastolic flow velocity; DL, dyslipidemia; DM, diabetes mellitus; DOAC, direct oral anticoagulant; DPP-4, dipeptidyl peptidase-4; ECG, electrocardiography; ECV, electrical cardioversion; EDV, end-diastolic velocity; EEG, electroencephalography; EF, ejection fraction; eGFR, estimated glomerular filtration rate; FV, flow volume (FV = time-averaged velocity x area x 60); HBF, hippocampal blood flow; HDL, high-density lipoprotein; HF, heart failure; HFrEF, heart failure with reduced ejection fraction; HR, heart rate; hs-TnT, high-sensitive troponin T concentration; HT, hypertension; i.v, intravenous; ICA, internal carotid artery; IHD, ischemic heart disease; LAd, left atrial diameter; LAv, left atrial volume; LAvi, left atrial volume index; LDL, low-density lipoprotein; LVdd, diastolic left ventricular diameter; LVds, systolic left ventricular diameter; LVEF, left ventricular ejection fraction; MAP, mean arterial pressure, MCAs, middle cerebral arteries; Med Ref RCAF, medical refractory rapidly conducted AF; MFV, mean flow velocity; MI, mitral insufficiency; MRI, magnetic resonance imaging; MV, mean velocity; NIH, National Institutes of Health; NIHSS, National Institutes of Health Stroke Scale; NIRS, near-infrared spectroscopy; NR, not reported; NT-proBNP, N-terminal pro brain natriuretic peptide concentration; NVAF, nonvalvular AF; NYHA, New York Heart Association; OACs, oral anticoagulants; PAD, peripheral artery disease; PAF, paroxysmal atrial fibrillation; PCI, percutaneous coronary intervention; PETCO2, end-tidal partial pressure of CO2; PFV, peak flow velocity; PI, pulsatility index; PM, pacemaker; PO2, partial pressure of oxygen; PRBC, packed red blood cells; PSV, peak systolic velocity; QUASAR-ASL, signal targeting with alternating radiofrequency (STAR) labeling of arterial regions arterial spin labeling; RAVLT, Rey Auditory Verbal Learning Test; rCBF, regional cerebral blood flow; RI, resistance index; SaO2, arterial oxygen saturation; sBP, systolic blood pressure; SctO2, cerebral tissue oxygen saturation; SFV, systolic flow velocity; SPECT, single-photon emission computed tomography; SR, sinus rhythm; SRS-NIRS, spatially resolved near-infrared spectroscopy; TCCS, transcranial color-coded duplex sonography; TCD, transcranial Doppler sonography; THI, tissue hemoglobin index; TIA, transient ischemic attack; VA, vertebral artery; VA-ECLS, venoarterial extracorporeal life support; VAF, valvular AF; VKA, vitamin K antagonist; vWF, von Willebrand factor; WBC, white blood cell; WHO, World Health Organization.

**Author, journal and year**	**Title**	**Aim**	**Type of intervention**	**Outcomes**	**Time to second measurement**	**Intervention and control groups: no. of participants**	**Measured baseline characteristics and possible confounders**	**Bias risk**
**Pre-post intervention studies**								
**Efimova et al. PACE 2012**[29]	Ablation and pacing: improving brain perfusion and cognitive function in patients with atrial fibrillation and uncontrolled ventricular rates	To determine if ablation and pacing improved brain perfusion and CBF in patients with medically refractory rapidly conducted AF	CA of AV-node, followed by PM implantation	a. Primary: rCBF (mL/100 g/min) measured by SPECT with 99mTc-hexamethylpropylene amine oxime tracer b. Secondary: neuropsychological tests, EF, CO, systolic output	3 months	Total: 32 AF →rate regularization (ventricular pacing): 17 SR controls: 15	Age, gender, AF etiology (valvular, ischemic heart disease, HT), NYHA class II-III.	Serious
**Gardarsdottir et al. Europace 2020**[26]	Improved brain perfusion after electrical cardioversion of atrial fibrillation	To test the hypothesis that blood flow to the brain is lowered in AF, resulting in decreased brain perfusion and CBF, and that successful cardioversion to SR leads to improvement in these parameters.	ECV	Cerebral perfusion by ASL MRI (mL/min) and total CBF (mL) by phase contrast MRI	>10 weeks	Total: 27 AF →SR: 17 AF →AF: 10	Age, height, weight, BMI, sBP, dBP, HR, ever smoker, HT, DL, coronary heart disease, PCI/CABG history, valvular dissease, other cardiac surgery or intervention, reduced cardiac contractility (EF < 55 %, clinical HF), other cardiac disease history, stroke, medication (anti-hypertensives, beta-blocker, warfarin, thrombin/Xa inhibitors, aspirin and antiplatelet, lipid lowering, diabetes medication, digoxin, class III antiarrhythmics)	Critical
**Hashimoto et al. Journal of Cardiovascular Electrophysiology 2023**[22]	Improved cerebral blood flow and hippocampal blood flow in stroke-free patients after catheter ablation of atrial fibrillation	To investigate the effect of CBF and HBF in stroke-free patients with PAF and non PAF.	CA	% changes from baseline CBF values (23.3 mL/100 g/min) by ASL MRI	3 months and 12 months	Total: 84 AF →SR: 23 SR →SR: 55 SR →AF: 2 AF →AF: 4	Age, gender, BMI, sBP, dBP, MAP, HR, CHF history, HT, DM, stroke history, CHA2DS2VASc score, LAd, LVEF, SV, CO, total cholesterol, HDL-cholesterol, LDL-cholesterol, triglyceride, eGFR, BNP, medications (ACE inhibitors and/or ARBs, beta-blockers, statins, oral anticoagulant, antiarrhytmic drugs) CA type (radiofrequency, pulmonary vein isolation, cavotricuspid ablation, superior vena cava isolation, box isolation)	Moderate
Kedžo et al. Life 2023 [25]	Association of electrical cardioversion with brain perfusion and cognitive function in patients with atrial fibrillation	To determine the association of persistent AF with brain perfusion and cognition, in comparison to control subjects and with regards to ECV	ECV	Regional brain perfusion by ASL MRI (metrics NR)	6 weeks	Total: 41 AF →SR: 8 AF →AF: 8 SR controls: 16	Age, gender, height, weight, BMI, sBP, dBP, HR, hemoglobin, haematocrit, creatinine, eGFR, blood glucose, albumin, NT-proBNP, hs-TNT, vWF, smoking, HT, DM, CKD, medication (anticoagulation, antiplatelets, beta-blocker, ACE inhibitor, statins)	Serious
**Petersen et al. Journal of Cerebral Blood Flow and Metabolism 1989**[43]	Cerebral blood flow before and after cardioversion of atrial fibrillation	To determine the CBF in patients with chronic AF of short duration before and after electrical cardioversion to SR	ECV	CBF by intravenous xenon-133 technique (initial slope index mL/100 g/min)	One day and 30 days	Total: 9 AF →SR: 9	Age, gender, thyroid status, sodium, potassium, creatinine, chest radiography, HT, congestive cardiomyopathy, renal failure, history of head injury or cerebrovascular disease, PCO2, HR, MAP, CO	Serious
**Porebska et al. Clinical Neurology and Neurosurgery 2007**[24]	Nonembolic, hemodynamic blood flow disturbances in the middle cerebral arteries in patients with paroxysmal atrial fibrillation without significant carotid stenosis	To evaluate whether patients with PAF without symptoms of cerebral ischemia or significant carotid stenosis have reduced blood flow through the MCAs, and to assess differences between blood flow through the MCAs during AF and after cardioversion.	i.v amiodarone	CBF by PFV, EDV and MFV of MCAs (cm/s) by TCD	Directly after conversion	Total: 65 AF →SR: 12 AF →AF: 18 SR controls: 35	Age, gender, HT, DM2, IHD, DL, sBP, dBP	Serious
**Saglietto et al. Europace 2021**[42]	Increased beat-to-beat variability of cerebral microcirculatory perfusion during atrial fibrillation: a near-infrared spectroscopy study	To monitor cerebral microcirculatory perfusion by SRS-NIRS to detect whether occurrence of critical hemodynamic events is reduced after SR restoration by ECV	ECV	Inter-beat THI variability by SRS-NIRS metrics	Directly after conversion	Total: 44 AF/AFL→ SR: 44	Age, gender, BMI, HT, DM, previous stroke/TIA, supra-aortic trunk stenosis, EHRA class, HF, CAD, CHA2DS2VASc score, HAS-BLED score, LVEF, LAvi, LVdd, medications (amiodarone, class IC, sotalol, beta-blocker, digoxin, aspirin, VKA, DOAC)	Serious
**Takahashi et al. JACC: Clinical Electrophysiology 2022**[27]	Increase in cerebral blood flow after catheter ablation of atrial fibrillation	To assess whether CA of AF led to increases in CBF and hippocampal volume	CA	CBF (mL) by 2D phase-contrast MR angiography and cerebral perfusion (mL/100 mL/min) as CBF/total brain volume	6 months	Total: 68 AF →SR: 57 AF controls: 11	Age, gender, BMI, type of AF (paroxysmal or nonparoxysmal), HT, DM, HF, prior thromboembolism, CHA2DS2VASc score, current smoking, former smoking, medications (oral anticoagulants, lipid-lowering agents, renin-angiotensin-aldosterone inhibitors), HR, eGFR, brain natriuretic peptide, LDL cholesterol, HDL cholesterol, LAd, LVEF	Serious
**Tatewaki et al. Journal of Clinical Medicine 2022**[28]	Impact of catheter ablation on brain microstructure and blood flow alterations for cognitive improvements in patients with atrial fibrillation: A pilot longitudinal study	To investigate whether CA of AF ablation can alter rCBF and brain microstructures, using multimodal MRI technique	CA	rCBF by QUASAR-ASL MRI, normalized ratios of gray matter/white matter	6 months	Total: 15 AF →SR: 8 SR controls: 7	Age, gender, HR, LAd, LVds, LVdd, EF, LAv, LAvi, CO, CI, serum BNP level, AF duration, CHADS2 score, medication use at baseline (anticoagulants, antiplatelets, anti-arrhythmics, antidepresssants)	Serious
**Totaro et al. The Italian Journal of Neurological Sciences 1993**[40]	Transcranial Doppler evaluation of cerebral blood flow in patients with paroxysmal atrial fibrillation	To assess CBF by TCD in 15 patients during and after an attack of PAF	i.v digitalis and oral quinidine or flecainide	CBF by MFV (cm/s) and PI (PI = SFV-DFV/MFV), by TCD	24 h	Total: 15 AF →SR: 15	Age, gender, first AF episode or previous PAF episodes, HR, MI, AI, signs of myocardial infarction on ECG, ICA stenosis	Serious
**Wutzler et al. Europace 2014**[23]	Improvement of cerebral oxygen saturation after successful electrical cardioversion of atrial fibrillation	To evaluate changes in cerebral oxygenation after ECV of AF, and to determine the correlation between changes in SctO2 and changes of the routinely measured parameters ABP, HR and SaO2	ECV	Change in SctO2 (%) by NIRS	∼30–40 min	Total: 30 AF →SR: 20 AF →AF: 10	Age, gender, BMI, AF type (paroxysmal or persistent), HT, CAD, structural heart disease, DM, PAD, LVEF, LAd, SctO2, SaO2, MAP, HR, baseline medication (Amiodarone, Beta-blocker, Digitoxin, ACE inhibitor, ARB, CCB, Statin, Diuretics, Aspirin, Oral anticoagulants), Anesthetics during CV (Midazolam, Etomidate)	Moderate
**Observational studies**								
**Vranken et al. Journal of Intensive Care Medicine 2020**[30]	Cerebral and limb tissue oxygenation during peripheral venoarterial extracorporeal life support	To assess the efficacy of cerebral and limb tissue oximetry during VA-ECLS	None	SctO2 (%) by NIRS	NA	Total: 20 AF →SR: 9 SR controls: 11	Age, gender, SaO2, arterial PO2, pulse oximetry (%), MAP, PRBC, fluid infusion (Ringer lactate or Gelofusin), limb temperature	Serious
**Cross-sectional studies**								
**Alosco et al. PACE 2015**[33]	Atrial fibrillation exacerbates cognitive dysfunction and cerebral perfusion in heart failure	To determine the impact of AF on cognitive function and cerebral perfusion among older adults with HF	None	a. Primary: CBF-V (cm/s) of MCA by TCD a. Secondary: neuropsychological tests	NA	Total: 187 HF AF: 60 HF SR: 127	Age, gender, years of education, LVEF, HT, DM2, sleep apnea, medications (anticoagulants, beta-blocker)	Serious
**Ameriso et al. Journal of Neuroimaging 1992**[37]	Atrial fibrillation, congestive heart failure, and the middle cerebral artery	To investigate potential nonembolic alterations in the cerebral circulation associated with AF and CHF	None	CBF-V (cm/s), pulsatility index and beat-to-beat variations of MCA by TCD	NA	Total: 47 Valvular AF: 13 Nonvalvular AF: 10 HF SR: 13 SR controls: 11	Age, gender, stroke risk factors (i.e hypertension, diabetes and CAD), MAP, CHF, ventricular cycle length, hematocrit, fibrinogen level, PCO2	Serious
**Babayiğit et al. Journal of Stroke and Cerebrovascular Diseases 2021**[36]	Assessment of cerebral blood flow velocities with transcranial Doppler ultrasonography in heart failure patients with reduced ejection fraction	To evaluate CBF velocities in patients with HFrEF with TCD	None	a. Primary: CBF-V (cm/s) of right and left MCA by TCD b. Secondary: EF	NA	Total: 72 HF AF: 9 HF SR: 37 SR controls: 26	Age, gender, height, weight, BMI, NYHA class, HT, sBP, dBP, HR, DM, stroke history, hyperlipidemia, smoking, creatinine, eGFR, hemoglobin, NT-proBNP	Critical
**Gardarsdottir et al. Europace 2018**[41]	Atrial fibrillation is associated with decreased total cerebral blood flow and brain perfusion	To evaluate and compare measured total CBF in the cervical arteries at the level of the skull base and estimated brain perfusion measured with MRI in individuals with and without AF at the time of imaging.	None	Total CBF (mL/min) and estimated brain perfusion in the entire brain (mL/100 g/ min) by phase-contrast MRI	NA	Total: 2291 AF diagnosis, in AF: 117 AF diagnosis, in SR: 78 SR controls: 2096	Age, gender, education, ever smoker, alcohol consumption, height, BMI, HT, sBP, MI, coronary heart disease, cholesterol, DM2, medications (anti-hypertensives, warfarin, aspirin), stroke, coronary artery calcium, total brain volume, intracranial volume, relative brain volume, AF type (paroxysmal vs persistent)	Serious
**Ide et al. Clinical Physiology 1999**[31]	Middle cerebral artery blood velocity during exercise in patients with atrial fibrillation	To study the difference in MCA Vmean in response to cycling between patients with AF and age-controlled subjects, and the MCA Vmean response to rhythmic handgrip in the AF patient group.	None	CBF by MCA MFV (cm/s) by TCD	NA	Total: 14 AF: 9 SR controls: 5	Age, gender, weight, height, blood pressure, HR, LAd, ST-changes on ECG, clinical presentation of AF, duration of AF, angina pectoris, prior AMI, HT history, NYHA classification, prior TIA, intermittent claudication, DM, hyperthyroidism, valvular heart disease, medication (digoxin, CCB, beta-blocker, ACE inhibitor, antithrombotic drugs)	Serious
**Junejo et al. Journal of Cerebral Blood Flow & Metabolism 2020**[32]	Neurovascular coupling and cerebral autoregulation in atrial fibrillation	To determine whether neurovascular coupling and cerebral autoregulation are impaired in AF.	None	CBF by MCA MFV (cm/s) by TCD	NA	Total: 83 AF: 30 HT SR: 29 SR controls: 24	Age, gender, waist/hip ratio, BMI, HT, DM2, HR, sBP, dBP, MAP, PETCO2, CHADS2 score, CHA2DS2VASc score, medications (beta-blockers, ACE inhibitor, CCB, diuretics, OACs, low-dose salicylates, digoxin, statins, biguanides, DPP-4 inhibitors, sulfonylureas, thiazolinediones)	Serious
**Junejo et al. Journal of the American College of Cardiology 2019**[5]	Impaired cerebrovascular reactivity in patients with atrial fibrillation	To test the hypothesis that CVR-CO2 is reduced in AF	None	CBF by MCA MFV (cm/s) by TCD	NA	Total: 92 AF: 31 HT SR: 31 SR controls: 30	Age, PETCO2, type of AF (paroxysmal vs persistent)	Serious
**Lavy et al. Stroke 1980**[44]	Effect of chronic atrial fibrillation on regional cerebral blood flow	To measure rCBF was measured in a group of patients with chronic AF and compare with flow data obtained in age-matched normal control subjects	None	rCBF by 1S3Xenon inhalation technique (initial slope index)	NA	Total: 81 AF: 31 SR controls: 50	Age, gender, AF etiology, ABP, HT, EEG	Critical
**Nakase et al, Alzheimeŕs Research & Therapy 2023**[38]	Impact of atrial fibrillation on the cognitive decline in Alzheimer's disease	To determine whether AF influences the severity of dementia and investigate any pathological difference based on brain imaging studies between dementia patients with and without AF	None	rCBF by N-isopropyl-p-123-I-iodoamphetamine SPECT data	NA	Total: 170 AF: 14 SR controls: 156	Age, gender, AD/aMCI, HT, DL, DM, dysthyroidism, CTR, sBP, dBP, eGFR, hemoglobin, WBC, CRP, homocysteine, vitamin B12, folic acid	Serious
**Porebska et al. Clinical Neurology and Neurosurgery 2008**[35]	Hemodynamic blood flow disturbances in the middle cerebral arteries in patients with atrial fibrillation during acute ischemic stroke	To evaluate whether blood flow in the MCAs in patients with AF during acute ischemic stroke is disturbed, and to assess if potential disturbances result from arrhythmia rather than a consequence of an embolus within the cerebral circulation	None	CBF by PVA, EDV and MFV of MCA (cm/s) by TCD	NA	Total: 180 AF: 90 SR controls: 90	Age, gender, HT, DM2, IHD, DL, sBP, dB, infarction on CT	Serious
**Su et al. Journal of the Formosan Medical Association 2017**[34]	Evaluation of cerebral blood flow in acute ischemic stroke patients with atrial fibrillation: A sonographic study	To use CCD and TCCS to evaluate the possible differences of cerebral hemodynamics between patients with AF and those with small vessel disease but without AF during acute ischemic stroke	None	PSV (cm/s), EDV (cm/s), MV (cm/s), RI and FV (mL/min) of ICA and MCA by CCD and TCCS. CBF (mL/min) determined as the sum of the FVs of the ICA and VA of both sides	NA	Total: 288 AF: 144 SR controls: 144	Age, gender, sBP, dBP, HR, hemoglobin, EF, small or large vessel occlusion, large atherosclerosis, cardioembolism, NIHSS	Moderate

#### 3.2.2 Comorbidities

In the observational study and in eight of the included cross-sectional studies, comorbidities were specifically reported [5], [30], [32], [33], [34], [35], [36], [37], [38]. The observational study focused on patients undergoing peripheral venoarterial extracorporeal life support [30]. Three cross-sectional studies included HF patients, comparing patients with or without AF [33], [36], or AF patients with and without HF, using HF without AF as a control [37]. Two studies included comparisons to SR controls with hypertension in addition to healthy SR controls [5], [32]. Two studies compared perfusion during acute ischemic stroke in patients with and without AF [34], [35]. One study included patients with Alzheimer’s disease or mild cognitive impairment, comparing those with and without AF [38].

#### 3.2.3 Cerebral perfusion measurements

The methods for measuring cerebral perfusion varied between studies (Table 1). Although positron emission tomography with the injection of [^15^O]-water radiotracer is regarded as the gold standard for CBF [39], none of the included studies used this method. The most frequently used methods were measurements of flow velocity with transcranial doppler sonography [5], [24], [31], [32], [33], [35], [36], [37], [40], or magnetic resonance imaging techniques [22], [25], [26], [27], [28], [41]. Other methods comprised near-infrared spectroscopy technology [23], [30], [42], color-coded carotid duplex sonography and transcranial color-coded duplex sonography [34], xenon-tracer techniques [43], [44] and single-photon emission computed tomography [29], [38].

#### 3.2.4 Quality assessment

According to the ROBINS-I/ROBINS-E assessment, risk of bias was rated as moderate in three studies, serious in 17 and critical in three (Table 1). The overall body of evidence assessed by GRADE [13] the main outcome was determined as low, due to risk of bias, large variation in effect sizes and measurement methods, and a somewhat selected AF population as the pre-post studies only included AF patients referred for intervention (Table 2).

**Table 2:** GRADE evidence table [13] Explanations: **a.** Majority of studies determined at serious risk of bias by ROBINS-I/ROBINS-E. **b.** Large variation in effect sizes and measurement methods is observed; however, the primary trend indicates reduced perfusion in patients with AF. **c.** Patients with AF selected for interventions may not be fully representative of the broader AF patient population. However, the cross-sectional studies encompass a more diverse range of AF patients, including some that specifically involve those with heart failure and AF. This broader inclusion ensures a wider representation of the AF patient population. **d.** Some p-values close to 0.05, or non-significant findings. **e.** Large variations in tests and measurement methods; some variations in results; however the primary trend suggests reduced cognitive function in patients with AF.

Certainty assessment							Certainty	Importance
№ of studies	Study design	Risk of bias	Inconsistency	Indirectness	Imprecision	Other considerations		
**Cerebral perfusion (main outcome)**								
23	Non-randomised studies	Serious^a^	Serious^b^	Serious^c^	Serious^d^	− All plausible residual confounding would reduce the demonstrated effect- Dose response gradient	⊕⊕○○ Low^a,b,c,d^	Critical

**Cognitive testing (secondary outcome)**.								
9	Non-randomised studies	Serious^a^	Serious^e^	Serious^c^	Serious^d^	− All plausible residual confounding would reduce the demonstrated effect − Dose response gradient	⊕⊕○○ Low^a,e,c,d^	Important

### 3.3 Primary outcomes

Out of 23 studies, 14 reported a difference in cerebral perfusion during AF compared to SR across all main outcome measurements (Table 3, Supplementary Material). Two studies found a difference in perfusion measurements pre and post intervention, but not when comparing pre-intervention AF patients to SR controls [24], [25]. Two studies investigated regional CBF in selected brain regions, both demonstrating increases in some areas while others remained unchanged [28], [29]. One study found improvements in cerebral perfusion only in patients < 65 years [44]. The exercise-based studies found differences at certain intensity levels, but not consistently across all time points during the exercise tests [31], [32]. One pre-post study found an increase in CBF after electrocardioversion only after adjustment for partial pressure of carbon dioxide [43]. Finally, the cross-sectional study comparing patients with Alzheimer’s disease or anamnestic mild cognitive impairment with and without AF, found no differences in cerebral perfusion metrics between the AF and SR groups [38]. Two studies investigated beat-to-beat variability of perfusion. One reported a change after conversion from AF to SR [42], and one observed a difference between patients in AF and control subjects in SR [37].

**Table 3:** **Outcomes.** Study groups are presented as rhythm status at baseline à follow-up cerebral perfusion measurements (pre-post studies), or rhythm status at time of assessment (cross-sectional studies). AF à SR indicates successful rhythm conversion and within-patient comparisons. AFà AF indicates unsuccessful conversion, (used as a control group when reported). In pre-post studies, SRà SR and SRà AF indicate patients with an AF diagnosis undergoing intervention, who were in SR during baseline (e.g., paroxysmal AF), and remained in SR or were in AF at follow-up. AF controls are AF patients without rhythm intervention (used as a control group when reported). SR controls are external control participants in SR; when applicable, these are specified by comorbidity (e.g., HF SR, HT SR). Abbreviations: AF, atrial fibrillation; CBF, cerebral blood flow; HF, heart failure; HT, hypertension; MCA, middle cerebral artery; MCAD, middle cerebral artery of damaged hemisphere; MCAI, middle cerebral artery of intact hemisphere; MFV, mean flow velocity; NR, not-reported; NVAF, nonvalvular atrial fibrillation; PCO₂, partial pressure of carbon dioxide; rCBF, regional cerebral blood flow; SctO₂, cerebral tissue oxygen saturation; SPECT, single-photon emission computed tomography; SR, sinus rhythm; THI, tissue hemoglobin index; VAF, valvular atrial fibrillation.

**Author, year**	**Effect metrics**	**Summary of findings**
**Pre-post intervention studies**		
**Efimova et al. 2012**[29]	rCBF (mL/100 g/min)	Sign. higher in SR vs AF group, all regions Sign. higher in superior frontal, both inferior frontal and left temporal regions post intervention No sign. difference in anterior or posterior parietal, right superior frontal, right temporal or occipital regions post intervention
**Gardarsdottir et al. 2020**[26]	a. Total CBF (mL) b. Brain perfusion (mL/min), whole brainc. Brain perfusion (mL/min) , grey matter	a. Sign. increase post ablation in AF →SR group.No sign. difference post intervention in SR →SR group b. Sign. increase post intervention in AF →SR group.No sign. differences post intervention in SR →SR group c. Sign. increase post intervention in AF →SR group.No sign. difference post intervention in SR →SR group
**Hashimoto et al. 2023**[22]	Differences (%) from control CBF (23.3 mL/100 g/min) a. Baselineb. Change post ablation c. 3 months vs 12 months	a. Sign. lower in AF ongoing vs AF not ongoing. Sign. lower in non-paroxysmal vs paroxysmal AF b. Sign. greater change in AF →SR group vs SR →SR group c. No sign. differences
**Kedžo et al. 2023**[25]	Global brain perfusion (metrics NR)	No sign. differences between AF patients pre intervention vs SR controls Sign. increase post intervention in AF →SR group
**Petersen et al. 1989**[43]	Initial slope index mL/100 g/min a. Unadjustedb. Adjusted for PCO2	a. No sign. difference post intervention b. Sign. increased post intervention
**Porebska et al. 2007**[24]	MFV (cm/s) of MCA a. Right MCAb. Left MCA	a. Sign. increased post intervention in AF →SR group.No sign. differences between AF patients pre intervention vs SR controls b. Sign. increased post intervention in AF →SR group.No sign. differences between AF patients pre intervention vs SR controls
**Saglietto et al. 2021**[42]	Inter-beat THI variability a. right tail, mr4b. left tail, ml4	a. Sign. decreased post intervention b. Sign. decreased post intervention
**Takahashi et al. 2022**[27]	a. Changes in CBF (mL/min), adjustedb. CBF % changec. Changes in brain perfusion (mL/100 mL/min), adjusted	a. Sign. greater increase in AF →SR group vs AF controls b. Sign. increase post intervention in AF →SR group c. Sign. greater increase in AF →SR group vs AF controls
**Tatewaki et al. 2022**[28]	rCBF ratios of whole gray matter/white matter	No sign. differences post intervention in AF →SR group post intervention No sign. differences in AF group vs SR controls Sign. higher in the left posterior cingulate gyrus post intervention in AF →SR group
**Totaro et al. 1993**[40]	MFV (cm/s) of MCA	Sign. increase post intervention
**Wutzler et al. 2014**[23]	Change in SctO2 (%) a. Left b. Right	a. Sign. greater change in AF →SR group vs SR →SR group b. Sign. greater change in AF →SR group vs SR →SR group
**Observational studies**		
**Vranken et al. 2020**[30]	SctO2 (%)	Sign. lower during AF compared to SR
**Cross-sectional studies**		
**Alosco et al. 2015**[33]	MFV (cm/s) of MCA	Sign. lower in HF AF vs HF SR
**Ameriso et al. 1992**[37]	a. MFV (cm/s) of MCA b. **%** beat-to-beat variations in MFV of MCA	a. NVAF: sign. lower vs SR controls VAF: sign. lower vs SR controls HF SR: sign. lower vs SR controls b. NVAF: sign. higher vs HF SR and SR controls VAF: Sign. higher vs HF SR and SR controls HF SR: Sign. higher vs SR controls
**Babayiğit et al. 2021**[36]	MFV (cm/s) of MCA a. Right MCAb. Left MCA	a. Sign. lower in HF AF vs HF SR. Sign. lower in HF AF vs SR controls b. Sign. lower in HF AF vs HF SR. Sign. lower in HF AF vs SR controls
**Gardarsdottir et al. 2018**[41]	a. Total CBF (mL/min), unadjustedb. Brain perfusion (mL/100 g/min), unadjusted	a. Sign. lower in AF vs SR b. Sign. lower in AF vs SR
**Ide et al. 1999**[31]	MFV (cm/s) of MCA a. At rest b. During intense cyclingc. % increase from rest to cycling	a. No sign. differences in AF vs SR at rest b. Lower in AF vs SR during intense cycling, p-value NR c. Sign. smaller % increase in AF vs SR
**Junejo et al. 2020**[32]	MFV (cm/s) of MCA a. Characteristics tableb. Baseline neurovascular coupling response testc. Change (%) at peak neurovascular coupling response test	a. Sign. lower in AF ongoing vs AF not ongoing Sign. lower in AF vs SR controls No sign. differences in AF vs HT SR b. No sign. differences c. Sign. higher in AF vs SR controls Sign. higher in AF vs HT SR
**Junejo et al. 2019**[5]	MFV (cm/s) of MCA	a. Sign. lower in AF vs HT SR Sign. lower in AF vs SR controls b. Sign. lower in AF ongoing vs AF not ongoing
**Lavy et al. 1980**[44]	Initial slope index a. 35–50 years b. 51–65 yearsc. 66–80 years	a. Sign. lower in AF vs SR b. Sign. lower in AF vs SR c. No sign. differences
**Nakase et al. 2023**[38]	rCBF reduction calculated using stereotactic extraction estimation SPECT, L/R	No sign. differences
**Porebska et al. 2008**[35]	MFV (cm/s) a. MCAD b. MCAIc. MCAD-MCAI	a. Sign. lower in AF vs SR b. Sign. lower in AF vs SR c. No sign. differences
**Su et al. 2017**[34]	a. MFV (cm/s) of MCA b. CBF (mL/min)	a. Sign. lower in AF vs SR b. Sign. lower in AF vs SR

### 3.4 Secondary outcomes and subgroup analysis

#### 3.4.1 Cognitive outcomes

Nine studies reported results from testing of cognitive function (Table 4). Among the intervention studies, one showed significantly improved test scores when comparing AF patients pre and post intervention [22], one observed no significant differences [25], and two found significant differences in some, but not all cognitive test scores [28], [29]. In two of the cross-sectional studies, AF groups had significantly less favourable cognitive test scores compared to SR controls [35], [38], and in two other there were no observed differences [34], [41]. One cross-sectional study found significant differences in some of the tests [33].

**Table 4:** **Secondary outcomes concerning neurocognitive testing.** Abbreviations: 3MS, Modified Mini-Mental State Examination; AVLT, Rey Auditory Verbal Learning Test; CES-D, Center for Epidemiologic Studies Depression Scale; CFT, Complex Figure Test; CVLT, California Verbal Learning Test; DCT, The Bourdon-Wiersma Dot Cancellation Test; DSST, Digit Symbol Substitution Test; HF, heart failure; LDFR, long-delay free recall; MMSE, Mini-Mental State Examination; MMSE-J, Japanese version of the Mini-Mental State Examination; NIHSS, National Institutes of Health Stroke Scale; PROMIS, Patient-Reported Outcomes Measurement Information System; RAVLT, Rey Auditory Verbal Learning Test; STAI, State-Trait Anxiety Inventory; TMT, Trail Making Test; WAIS, Wechsler Adult Intelligence Scale, Fourth Edition

Author, year	Neurocognitive testing metrics	Summary of findings
Pre-post intervention studies		
Efimova et al. 2012 [29]	AVLT after first submission, 30 min and in eight reduplications, CFT after first submission and 30 min; DCT time and mistakes; Digit span forward backward numbers; Digit symbol test signs; TMT parts A and B; Token test score	Sign. improved scores post intervention in 10 of 12 tests and sign. less favorable scores in all tests compared to SR group
Hashimoto et al. 2023 [22]	MMSE	Sign. improved scores post intervention in all patients
Kedžo et al. 2023 [25]	PROMIS	No sign. differences
Tatewaki et al. 2022 [28]	CES-D; MMSE; RAVLT Forgetting; RAVLT Learning; RAVLT Recognition; RAVLT Total recall; STAI-Y1; STAI-Y2; TMT-A (s); TMT-B (s); WAIS digit span; WAIS digit symbol	Sign. improved scores post intervention in 4 of 13 tests and sign. less favorable scores in 3 of 13 tests in AF group compared to SR group
Cross-sectional studies		
Alosco et al. 2015 [33]	3MS; Animal fluency; Boston naming test; CVLT-II LDFR; CVLY-II recognition hits; Digit symbol coding; Frontal assessment battery; TMT-A; TMT-B	Sign. less favorable scores in 2 of 9 tests in HF AF patients compared to HF SR patients
Gardarsdottir et al. 2018 [41]	DSST, MMSE	No sign. differences
Nakase et al. 2023 [38]	MMSE-J	Sign. less favorable scores in AF group compared to SR group
Porebska et al. 2008 [35]	NIHSS	Sign. less favorable scores in AF group compared to SR group
Su et al. 2017 [34]	Barthel index, modified Rankin Scale, NIHSS	No sign. differences

#### 3.4.2 Heart failure

Both studies comparing cerebral perfusion in HF patients with and without AF concluded that HF patients in AF had impaired cerebral perfusion compared to HF patients in SR [33], [36]. One of these included only HF patients with New York Heart Association Functional Classification (NYHA) classes II-IV, with a mean LVEF of 37.08 % (± 15.72) in HF patients with AF and 41.84 % (± 13.55) in HF patients without AF (χ2 /t = -2.02, p < 0.05). No significant difference in beta-blocker use between the two groups was reported [33]. Additional medication use was not described [33]. The other study included only HF patients with reduced LVEF (HFrEF), all in NYHA classes II-IV [36]. In this study, mean LVEF was 21 % (± 3.12) in the HFrEF with AF group, and 19.92 % (± 3.99) in those without AF (p = 0.045) [36]. No information on use of medication was available. The third HF study compared cerebral perfusion in AF patients with and without HF, and found a higher pulsatile index in the middle cerebral artery of AF patients with HF than those without, but no differences in peak, mean and diastolic flow velocities [37]. The authors also reported a significantly lower mean blood flow velocity in AF patients compared to HF patients in SR and to healthy SR controls [37]. This study included HF patients with NYHA classes II-IV [37]. There were no reports on LVEF or medication [37]. Finally, two of these three HF subgroup studies compared CBF velocities in HF patients in SR to healthy SR controls [36], [37]. Both reported significantly lower velocities in HF patients compared to healthy controls [36], [37].

## 4 Discussion

We performed a systematic review of existing literature to assess the evidence from clinical studies on AF as a cause of impaired cerebral perfusion which has been associated with cognitive dysfunction independently of thromboembolic events [2]. In contrast to previous studies, we included patients with HF, in whom AF might have greater effects on the circulation and cerebral perfusion. We identified 23 relevant studies, of which 11 were intervention studies, but none were randomized. Twenty-two studies concluded that AF can be an independent cause of impaired cerebral perfusion while one study found no such effect. One study reported an age-dependent effect [44]. Three studies reported results from patients with HF, with two concluding that HF patients with AF had lower cerebral perfusion compared to HF patients in SR.

Our review of existing literature indicates an association between AF and reduced cerebral perfusion. This aligns with another recently conducted systematic review on interventional studies which concluded that restoring SR positively impacts cerebral perfusion [4]. The added value of our review is inclusion not only of interventional studies, but also cross-sectional studies comparing AF patients to SR controls, and studies specifically focusing on HF comorbidity. The overall assessment of the evidence from these studies is in support of an effect of AF on cerebral perfusion, but most of the studies included had a high risk of bias. The pre-post studies had a high risk of selection bias, while the cross-sectional studies had other methodologic limitations that restricted their generalizability.

A key limitation in synthesizing findings is the lack of consistency in how cerebral perfusion is defined and measured. While some studies directly measured CBF using standardized units [22], [25], [26], [27], [28], [29], [38], [41], [43], [44], others used surrogate markers such as oxygen saturation [23], [30], [42] or regional flow velocity [5], [24], [31], [32], [33], [34], [35], [36], [37], [40]. This heterogeneity limits comparability and highlights the need for more standardized definitions and methodologies in future research.

The studies in our review were mostly focused on differences in cerebral perfusion in the acute to short term [22], [23], [24], [25], [26], [27], [28], [29], [40], [42], [43]. The long-term effects from AF on cerebral perfusion independent of thromboembolic events therefore remains unknown. Following catheter ablation for AF, Takahashi et al. observed that CBF continued to increase for six months [27], Tatewaki et al. measured increased rCBF in the left posterior cingulate after 12 months [28], and Hashimoto et al. showed that the increase in CBF observed at three months maintained after 12 months [22]. This suggests that the impact on cerebral perfusion extends beyond immediate hemodynamic changes after restoring SR. Future long-term follow-up studies are needed to determine the long-term effects of AF on cerebral perfusion.

### 4.1 Impaired cerebral perfusion as a cause of cognitive dysfunction in AF

Impaired cerebral perfusion has been suggested to cause cognitive dysfunction in AF [3], [4]. The results of cognitive testing in the studies included in our review does not provide clear evidence. Differences in cognitive test scores between patients with AF and SR support an association [33], [35], [38], as do improvements in cognitive performance following AF conversion [22], [28], [29]. However, two cross-sectional and one pre-post study reported no significant differences in cognitive testing during AF versus SR [25], [34], [41]. In their review of interventions for AF, Rasti et al. also found divergent results, with two of four studies showing an improvement of cognitive function post intervention [4]. However, in the studies showing a non-significant change, time interval to investigation was shorter than what is recommended for assessing long‐term neuropsychological functioning, and repeated cognitive testing within a limited timeframe may introduce bias through training effects [4]. One of these studies was also among those in our review that showed no difference [25]. The other two studies in our review with similar findings were cross-sectional comparisons between AF patients and SR controls.

Further studies should address the cumulative impact of AF on cerebral perfusion, rather than a simple AF versus SR comparison. Randomized trials are limited by ethical concerns and recurrence of AF, making long-term rhythm control studies difficult. Future research should prioritize large, longitudinal observational studies with continuous rhythm monitoring and standardized perfusion assessment. These designs must control for key confounders such as age, comorbidities, and medication use. Such data would enable more reliable *meta*-analyses and clarify the long-term cerebrovascular consequences of AF.

### 4.2 Subgroup analyses in patients with heart failure

In the context of impaired cerebral perfusion as a result of variable stroke volume and cardiac output, patients with impaired cardiac function due to HF are of special interest. When measuring brain perfusion in patients with HF, multiple brain areas show regional hypoperfusion [6]. CBF has in fact been observed to decrease by 14–30 % in severe cases of HF, a reduction suggested to be associated with reduced cardiac output [7]. As an additional aspect compared to previous review articles, we therefore included the results from studies on patients with AF and HF [33], [36], [37]. These studies indicate that both AF and HF individually impair cerebral perfusion, and that reductions are more pronounced in patients with both conditions. However, the only study directly comparing AF patients with and without HF did not confirm a synergistic effect [37]. Additionally, data on factors influencing CBF, such as LVEF categories, NYHA class, and HF medical therapy, were sparse and varied across studies. The contribution of reduced cardiac function for cerebral perfusion in the setting of AF is therefore still not clear, and further studies are warranted to validate the observed outcomes.

### 4.3 Proposed pathophysiological explanations supporting the observed associations

Although the findings of this review support a difference in cerebral perfusion during AF compared to SR, the underlying mechanisms remain contentious. When assessing hemodynamics in computational models, variability in AF has been shown to cause notable cerebral hemodynamic disturbances [45], [46]. Indeed, two of the included studies in this review documented beat-to-beat variability in cerebral perfusion metrics, providing additional support for this theory [37], [42]. However, the measurements in these studies are indirect, and additional research is required to confirm and extend these findings. An alternative hypothesis suggests that AF directly impairs cerebral autoregulation [34], [40]. In support of this, one of the included studies in this review explored neurovascular coupling and concluded that AF adversely affects this process [32]. These findings are in accordance with experimental findings from canine models, where AF induction resulted in a marked reduction in cerebrovascular reserve, with only partial recovery observed following the reestablishment of SR [47].

### 4.4 Implications for clinical practice

Treatment of AF aims to alleviate symptoms and reduce stroke risk. A causal link between AF and cognitive dysfunction would reinforce the importance of rhythm control in AF management. A deeper understanding of the mechanisms linking AF to cerebral perfusion is essential for establishing causality. Current data suggest that AF may negatively impact cerebral perfusion, although with significant caveats. This seems to add further support for rhythm control, perhaps especially in individuals with other risk factors for cognitive dysfunction.

## 5 Conclusions

Current evidence suggests that AF may impair cerebral perfusion, potentially contributing to cognitive decline. However, the evidence remains weak due to variations in study designs and measurement techniques, and the mechanism for cognitive impairment remains unresolved. Further long-term clinical and mechanistic studies are needed to determine if prevention of cognitive dysfunction is an argument for rhythm control in patients with AF.

## 6 Lay summary

AF may reduce blood flow to the brain and contribute to cognitive problems, especially in patients who also have HF.

• In this systematic review of 23 studies, most found that AF was associated with lower brain perfusion compared to normal heart rhythm, and five of nine studies reported poorer cognitive test results in people with AF.
• Some studies suggested that people with both AF and HF may experience even lower brain blood flow.

## CRediT authorship contribution statement

**Pernille Borch:** Writing – original draft, Visualization, Resources, Methodology, Investigation, Formal analysis, Data curation, Conceptualization. **Stine Aagaard-Nilsen:** Writing – review & editing, Validation, Investigation, Formal analysis, Data curation. **Mathis Korseberg Stokke:** Writing – review & editing, Supervision, Methodology, Conceptualization. **Erik Lyseggen:** Writing – review & editing, Supervision, Project administration, Methodology, Investigation, Conceptualization.

## Declaration of competing interest

The authors declare that they have no known competing financial interests or personal relationships that could have appeared to influence the work reported in this paper.
