# Supplementary material for: Effects of atrial fibrillation on cerebral perfusion and cognitive function: A systematic review
Source: Int J Cardiol Heart Vasc. 2026 Feb 20;63:101891. doi: 10.1016/j.ijcha.2026.101891 (PMC12945524; doi:10.1016/j.ijcha.2026.101891)
Supplement: Supplementary Data 1 [file mmc1.docx]

# Supplementary material

| **Author, year** | **Concludes with positive effect of AF on perfusion** | **Effect metrics** | **Effect measure** | **Effect size** |
| --- | --- | --- | --- | --- |
| **Pre-post intervention studies** | | | | |
| **Efimova et al. 2012^29^** | Yes - SR vs AF group, all regions Yes - Left superior frontal, both inferior frontal and left temporal regions post intervention No - anterior or posterior parietal, right superior frontal, right temporal or either occipital regions post intervention | rCBF (mL/100g/min), mean ± SD | **Anterior parietal, left:** 44.7 ± 4.3 (AF🡪SR pre)  vs 44.6 ± 4.8 (AF🡪SR post) (p=0.432) vs 50.4 ± 2.6 (SR) (p=0.013) **Anterior parietal, right:** 46.5 ± 4.1 (AF 🡪SR pre)  vs 48.5 ± 5.1 (AF🡪SR post) (p=0.051) vs 51.3 ± 2.8 (SR) (p=0.031) **Posterior parietal, left:** 48.6 ± 6.8 (AF🡪SR pre)  vs 50.5 ± 5.7 (AF🡪SR post) (p=0.056) vs 56.9 ± 2.1 (SR) (p=0.024) **Posterior parietal, right:** 51.1 ± 4.4 (AF🡪SR pre)  vs 52.9 ± 5.2 (AF🡪SR post) (p=0.078) vs 55.4 ± 2.7 (SR) (p=0.032) **Superior frontal, left:** 47.3 ± 5.7 (AF🡪SR pre)  vs 49.7 ± 5.2 (AF🡪SR post) (p=0.007) vs 52.3 ± 2.6 (SR) (p=0.026) **Superior frontal, right:** 49.6 ± 6.3 (AF🡪SR pre)  vs 50.2 ± 6.1 (AF🡪SR post) (p=0.064) vs 53.4 ± 2.5 (SR) (p=0.028) **Inferior frontal, left:** 43.9 ± 8.1 (AF🡪SR pre)  vs 46.8 ± 5.4 (AF🡪SR post) (p=0.049) vs 54.5 ± 2.8 (SR) (p=0.003) **Inferior frontal, right:** 43.3 ± 9.2 (AF🡪SR pre)  vs 48.3 ± 7.3 (AF🡪SR post) (p=0.010) vs 55.4 ± 2.4 (SR) (p=0.002) **Temporal, left:** 49.1 ± 6.1 (AF🡪SR pre)  vs 52.0 ± 5.5 (AF🡪SR post) (p=0.005) vs 55.4 ± 2.8 (SR) (p=0.032) **Temporal, right:** 52.5 ± 5.4 (AF🡪SR pre)  vs 53.3 ± 4.7 (AF🡪SR post) (p=0.054) vs 56.4 ± 2.8 (SR) (p=0.037) **Occipital, left:** 51.8 ± 7.8 (AF🡪SR pre)  vs 51.9 ± 7.0 (AF🡪SR post) (p=0.345) vs 58.4 ± 2.9 (SR) (p=0.011) **Occipital, right:** 53.2 ± 7.3 (AF🡪SR pre)  vs 56.7 ± 9.6 (AF🡪SR post) (p=0.050) vs 58.1 ± 2.7 (SR) (p=0.019) | AF🡪SR pre vs post: −0,2 % to 10,4 % increase post  AF pre vs SR: 6,9 % to 21,8 % higher in SR |
| **Gardarsdottir et al. 2020^26^** | Yes | a. Total CBF (mL), mean ± SD b. Brain perfusion (mL/min), whole brain, mean ± SD  c. Brain perfusion (mL/min), grey matter, mean ± SD | a. 555.2 ± 29.2 (AF🡪SR pre) vs 613.8 ± 29.5 (AF🡪SR post), p<0.05 586.8 ± 38.2 (AF🡪AF pre) vs 566.0 ± 39.9 (AF🡪AF post), p=0.52  b. 36.3 ± 1.9 (AF🡪SR pre) vs 41.2 ± 1.9 (AF🡪SR post), p<0.001  34.4 ± 2.6 (AF🡪AF pre) vs 32.8 ± 2.6 (AF🡪AF post), p=0.36  c. 39.9 ± 2.3 (AF🡪SR pre) vs 45.5 ± 2.3 (AF🡪SR post), p<0.001 37.8 ± 2.9 (AF🡪AF pre) vs 35.9 ± 3.0 (AF🡪AF post), p=0.34 | a. AF🡪SR: 9.5 % increase post  AF🡪AF : 3.7 % decrease post  b. AF🡪SR: 11.9 % increase post  AF🡪AF : 4.9 % decrease post  c. AF🡪SR: 12.3 % increase post  AF🡪AF : 5.3 % decrease post |
| **Hashimoto et al. 2023^22^** | Yes | Differences (%) from control CBF (23.3 mL/100 g/min) ± SD (range) a. Baseline b. Change post ablation  c. 3 months vs 12 months | a. 80 ± 24 % (AF ongoing) vs 101 ± 32 % (AF not ongoing), p<0.01 100 ± 32 % (paroxysmal AF) vs 86 ± 28 % (non-paroxysmal AF), p=0.04  b. Overall median change: +17.5 %, p NR 23.9 % (-4.1-33.7) (AF🡪SR) vs 2.2 % (−13.0 to 12.8) (SR🡪SR), p<0.01  c. 104 ± 33 % (paroxysmal AF 3 months) vs 102 ± 32 % (paroxysmal AF 12 months), p=0.81.  99 ± 32 % (non-paroxysmal AF 3 months) vs 102 ± 26 % (non-paroxysmal AF 12 months), p=0.72 | a. 20.8 % lower in AF ongoing vs AF not ongoing.  14.0 % lower in non-paroxysmal vs paroxysmal AF  b. Overall median: 17% increase post ablation  986.4 % greater change in AF🡪SR vs SR🡪SR  c. 2.0 % higher at 3 vs 12 months in paroxysmal AF  3.0 % higher at 12 vs 3 months in non-paroxysmal AF |
| **Kedžo et al. 2023^25^** | Yes - global brain perfusion pre vs post intervention No - global brain perfusion in AF patients pre intervention vs SR controls | Global brain perfusion, mean ± SD (metrics NR) | 297 ± 24 (pre) vs 328 ± 37 (post), p=0.008 | 10.4 % increase post |
| **Petersen et al. 1989^43^** | Yes - corrected for PCO2 No - uncorrected | Initial slope index mL/100g/min, median (range) a. Unadjusted b. Adjusted for PCO2 | a. 35.8 (24.1-44.1) (pre) vs 37.1 (31.9-52.7) (1d post) and 39.4 (29.1-55.7) (30d post), ns b. 35.5 (24.1-44.1) (pre) vs 40.3 (30.9-54.9) (1d post) and 46.7 (29.9-54.3) (30d post), Tate-Clelland's test: p=0.009, Friedman's test: p=0.018 | a. 3.6 % increase 1d post, 10.1 % increase 30d post  b. 13.5 % increase 1d post, 31.5 % increase 30d post |
| **Porebska et al. 2007^24^** | Yes - MFV of MCAs pre vs post intervention No - MFV of MCAs in AF patients pre intervention vs SR controls | MFV (cm/s) of MCA, median (range) a. Right MCA b. Left MCA | a. 32.6 (19.3–48.0) (pre) vs 42.65 (26.0–59.7) (post), p=0.008  b. 34.7 (15.4-56.3) (pre) vs 43.35 (21.0-64.9) (post), p=0.004 | a. 30.8 % increase post  b. 24.9 % increase post |
| **Saglietto et al. 2021^42^** | Yes | Inter-beat THI variability a. right tail, mr4 b. left tail, ml4 | a. 7.41×10^–9^ (2.26×10^-9^, 2.04×10^-8^) (pre) vs 1.94×10^–9^ (4.32×10^–10^, 6.79×10^-9^) (post) (p<0.001)  b. 4.33×10^–9^ (1.03×10^–9^, 1.10×10^–8^) (pre) vs 1.30×10^–9^ (5.24×10^–10^, 5.37×10^–9^) (post) (p=0.047) | a. 73.8 % decrease post  b. 70.0 % decrease post |
| **Takahashi et al. 2022^27^** | Yes | a. Changes in CBF (mL/min), adjusted mean (95 % CI) b. CBF % change c. Changes in brain perfusion (mL/100mL/min), adjusted mean (95 % CI) | a. +39.26 (4.52-74.00) (AF🡪SR) vs -34.86 (-96.61-26.89) (AF controls), p=0.01  b. +13.7 % (AF🡪SR pre vs post), p<0.0001  c. +3.78 (0.60-6.96) (AF🡪SR) vs -3.02 (-8.62 to 2.57) (AF controls), p=0.009 | a. 212.6 % greater increase in ablated vs AF controls  b. 13.7 % increase post  c. 225.2 % greater change in AF🡪SR vs AF controls |
| **Tatewaki et al. 2022^28^** | No - Whole cerebral grey matter CBF ratio (AF🡪SRpre vs post intervention and AF group vs SR group)  Yes - rCBF in the left posterior cingulate gyrus (AF group pre vs post intervention) | rCBF ratios of whole gray matter/white matter, mean ± SD | 1.59 ± 0.07 (pre) vs 1.64 ± 0.07 (post), p=0.61 | 3.1 % increase post |
| **Totaro et al. 1993^40^** | Yes | MFV (cm/s) of MCA, mean ± SD | 48.8 ± 16 (pre) vs 55.1 ± 10.9 (post), p<0.05 | 12.9 % increase post |
| **Wutzler et al. 2014^23^** | Yes | Change in SctO2 (%), mean ± SD  a. Left  b. Right | a. Left: +4.27 ± 3.56 (AF🡪SR) vs -0.38 ± 2.4 (AF🡪AF), p<0.001  b. Right: +3.25 ± 2.5 (AF🡪SR) vs -0.13 ± 0.52 (AF🡪AF), p=0.001 | a. 1223.7 % greater change (AF🡪SR vs AF🡪AF)  b. 2600.0 % greater change (AF🡪SR vs AF🡪AF) |
| **Observational studies** | | | | |
| **Vranken et al. 2020^30^** | Yes | SctO2 (%), mean ± SD | 61 % ± 11 % (SR) vs 51 % ± 10 % (AF), p<0.001 | 16.4 % lower in AF vs SR |
| **Cross-sectional studies** | | | | |
| **Alosco et al. 2015^33^** | Yes | MFV (cm/s) of MCA, mean | 37.00 (HF AF) vs 43.41 (HF SR), p<0.01 | 14.8 % lower in HF AF vs HF SR |
| **Ameriso et al. 1992^37^** | Yes | a. MFV (cm/s) of MCA, mean ± SD b. **%** beat-to-beat variations in MFV of MCA | a. 34.5 ± 15.0 (NVAF)*, 36.9 ± 13.5 (VAF)*, 35.2 ± 10.1 (CHF)*, 56.6 ± 26.9 (SR controls), * = p<0.005 vs SR controls  b. 17.0 ± 14.2 (NVAF)**, 16.6 ± 14.9 (VAF)**, 3.4 ± 3.3 (CHF)*, 1.7 ± 1.8 (SR controls), * = p<0.005 vs SR controls, ** = p<0.005 vs SR controls and CHF | a. NVAF: 39.0 % lower vs SR controls  VAF: 34.8 % lower vs SR controls  CHF: 37.8 % lower vs SR controls  b. NVAF: 900.0 % higher vs SR controls  VAF: 876.5 % higher vs SR controls  CHF: 100.0 % higher vs SR controls |
| **Babayiğit et al. 2021^36^** | Yes | MFV (cm/s) of MCA, mean ± SD a. Right MCA b. Left MCA | a. 38.55 ± 16.63 (HF AF)  vs 50.91 ± 15.64 (HF SR), p=0.04 vs 56.41 ± 10.75 (SR controls), p=0.01  b. 37.8 ± 12.02 (HF AF)  vs 49.95 ± 14.64 (HF SR), p=0.03 vs 57.15 ± 12.47 (SR controls), p<0.001 | a. HF AF 24.3 % lower vs HF SR  HF AF 31.7 % lower vs SR controls  b. HF AF 24.3 % lower vs HF SR  HF AF 33.9 % lower vs SR controls |
| **Gardarsdottir et al. 2018^41^** | Yes | a. Total CBF (mL/min), unadjusted b. Brain perfusion (mL/100g/min), unadjusted | a. 472.1 (persistent AF) vs 541.0 (SR), p<0.001  b. 46.4 (persistent AF) vs 52.8 (SR), p<0.001 | a. 12.7 % lower in persistent AF vs SR  b. 12.1 % lower in persistent AF vs SR |
| **Ide et al. 1999^31^** | No - at rest Yes - increase during intense cycling | MFV (cm/s) of MCA, median (range) a. At rest b. During intense cycling c. % increase from rest to cycling | a. 43 (39-56) (AF) vs 52 (40-68) (SR), p>0.05  b. 51 (40-78) (AF) vs 62 (50-81), p-value NR  c. 9 % (AF) vs 23 % (SR), p<0.05 | a. 17.3 % lower in AF vs SR at rest  b. 17.7 % lower in AF vs SR during intense cycling  c. 60.9 % smaller % increase in AF vs SR |
| **Junejo et al. 2020^32^** | Yes - characteristic tables and peak neurovascular coupling response No - baseline during functional assessments of neurovascular coupling response | MFV (cm/s) of MCA a. Characteristics table b. Baseline neurovascular coupling response test c. Change (%) at peak neurovascular coupling response test | a. 44 ± 11 (AF ongoing) vs 59 ± 10 (AF not ongoing), p<0.01 51 ± 13 (all AF)  vs 62 ± 14 (SR controls), p<0.05  vs 54 ± 11 (HT SR), ns   b. 49 ± 12 (AF)  vs 60 ± 15 (SR controls), ns vs 55 ± 12 (HT SR), ns   c. 19 ± 6 (AF)  vs 11 ± 7 (SR controls), p<0.05 vs 12 ± 3 (HT SR), p<0.05 | a. 25.4 % lower in AF ongoing vs AF not ongoing  17.7 % lower in all AF vs SR controls  5.6 % lower in all AF vs HT SR  b. 18.3 % lower in AF vs SR controls  10.9 % lower in AF vs HT SR  c. 72.7 % higher in AF vs SR controls  58.3 % higher in AF vs HT SR |
| **Junejo et al. 2019^5^** | Yes | MFV (cm/s) of MCA, mean ± SD | a. 51.0 ± 12.9 (all AF) vs 53.1 ± 11.1 HT SR) and 60.9±12.9 (SR), p=0.006  b. 31.1 ± 8.7 (AF ongoing) vs 59.2 ± 10.5 (AF not ongoing), p<0.001 | a. 4.0 % lower in all AF vs HT SR  16.3 % lower in all AF vs SR  b. 47.5 % lower in AF ongoing vs AF not ongoing |
| **Lavy et al. 1980^44^** | Yes - 35-50 years and 51-65 years No - 66-80 years | Initial slope index, mean ± SD a. 35-50 years b. 51-65 years c. 66-80 years | a. 43.6 ± 7.2 (AF) vs 52.9 ± 7.1 (SR), p<0.01  b. 42.8 ± 7.1 (AF) vs 49.4 ± 6.8 (SR), p<0.01  c. 42.5 ± 6.2 (AF) vs 45.0 ± 6.0 (SR), ns | a. 17.6 % lower in AF vs SR  b. 13.4 % lower in AF vs SR  c. 5.6 % lower in AF vs SR |
| **Nakase et al. 2023^38^** | No | rCBF reduction calculated using stereotactic extraction estimation SPECT, L/R, mean ± SD (metrics NR) | Superior frontal gyrus: 71.6 ± 25.3 / 72.9 ± 28.0 (AF) vs 64.0 ± 27.6 / 62.6 ± 28.8 (SR), p=0.3595 / 0.2350  Medial frontal gyrus: 75.7 ± 18.8 / 75.9 ± 21.9 (AF) vs 66.7 ± 25.1 / 66.5 ± 26.1 (SR), p=0.2300 / 0.2304  Precuneus: 71.3 ± 32.4 / 74.8 ± 33.4 (AF) vs 66.3 ± 31.3 / 68.3 ± 31.7 (SR), p=0.5970 / 0.4989  Thalamus: 86.9 ± 24.5 / 89.0 ± 26.9 (AF) vs 70.0 ± 34.1 / 80.9 ± 29.3 (SR), p=0.0958 / 0.3573  Parahippocampal gyrus: 82.1 ± 16.4 / 85.0 ± 16.1 (AF) vs 73.5 ± 29.9 / 74.5 ± 29.2 (SR), p=0.3273 / 0.2194  Posterior cingulate gyrus: 83.5 ± 26.8 / 87.2 ± 23.9 (AF) vs 78.4 ± 25.6 / 74.9 ± 26.6 (SR), p=0.5070 / 0.1223 | 6.5 % to 24.1 % higher in AF vs SR |
| **Porebska et al. 2008^35^** | Yes | MFV (cm/s) min-max, mean ± SD (range) a. MCAD b. MCAI c. MCAD-MCAI | a. 26.99 ± 13.05 (12.2-68.3) (AF) vs 40.05 ± 18.69 (14.0-86.0) (SR), p<0.0001  b. 27.06 ± 13.79 (11.8-87.5) (AF) vs 38.12 ± 17.15 (12.7–105.3) (SR), p<0.0001  c. −0.01 ± 12.15 (-45.5-37.4) (AF) vs 1.93 ± 15.51 (−34.0–46.4) (SR), ns | a. 32.6 % lower in AF vs SR  b. 29.0 % lower in AF vs SR  c. 100.5 % lower in AF vs SR |
| **Su et al. 2017^34^** | Yes | a. MFV (cm/s) of MCA, mean ± SD b. CBF (mL/min) | a. 49 ± 19 (AF) vs 59 ± 29 (SR), p<0.001  b. 582 ± 150 (AF) vs 654 ± 160 (SR), p<0.001 | a. 16.9 % lower in AF vs SR  b. 11.0 % lower in AF vs SR |

**Supplementary table of outcomes.** Study groups are presented as rhythm status at baseline 🡪 follow-up cerebral perfusion measurements (pre-post studies), or rhythm status at time of assessment (cross-sectional studies). AF 🡪 SR indicates successful rhythm conversion and within-patient comparisons. AF🡪AF indicates unsuccessful conversion, (used as a control group when reported). In pre-post studies, SR🡪SR and SR🡪AF indicate patients with AF diagnosis undergoing intervention, who were in SR during baseline (e.g., paroxysmal AF), and remained in SR or were in AF at follow-up. AF controls are AF patients without rhythm intervention (used as a control group when reported). SR controls are external control participants in SR; when applicable, these are specified by comorbidity (e.g., HF SR, HT SR). Abbreviations: AF, atrial fibrillation; CBF, cerebral blood flow; CHF, congestive heart failure; HF, heart failure; HFrEF, heart failure with reduced ejection fraction; HT, hypertension; MCA, middle cerebral artery; MCAD, middle cerebral artery of damaged hemisphere; MCAI, middle cerebral artery of intact hemisphere; MFV, mean flow velocity; NR, not reported; ns, not significant; NVAF, non-valvular atrial fibrillation; PCO₂, partial pressure of carbon dioxide; rCBF, regional cerebral blood flow; SctO₂, cerebral tissue oxygen saturation; SPECT, single-photon emission computed tomography; SR, sinus rhythm; THI, tissue hemoglobin index; UC, unsuccessful conversion; VAF, valvular atrial fibrillation.
